# Gene Expression Profiling Identifies IRF4-Associated Molecular Signatures in Hematological Malignancies

**DOI:** 10.1371/journal.pone.0106788

**Published:** 2014-09-10

**Authors:** Ling Wang, Zhi Q. Yao, Jonathan P. Moorman, Yanji Xu, Shunbin Ning

**Affiliations:** 1 Center for Inflammation, Infectious Diseases and Immunity, Quillen College of Medicine, East Tennessee State University, Johnson City, Tennessee, United States of America; 2 HIV/HCV Program, James H. Quillen VA Medical Center, Johnson City, Tennessee, United States of America; 3 Shaun and Lilly International, LLC, Collierville, Tennessee, United States of America; University of Nebraska - Lincoln, United States of America

## Abstract

The lymphocyte-specific transcription factor Interferon (IFN) Regulatory Factor 4 (IRF4) is implicated in certain types of lymphoid and myeloid malignancies. However, the molecular mechanisms underlying its interactions with these malignancies are largely unknown. In this study, we have first profiled molecular signatures associated with IRF4 expression in associated cancers, by analyzing existing gene expression profiling datasets. Our results show that IRF4 is overexpressed in melanoma, in addition to previously reported contexts including leukemia, myeloma, and lymphoma, and that IRF4 is associated with a unique gene expression pattern in each context. A pool of important genes involved in B-cell development, oncogenesis, cell cycle regulation, and cell death including BATF, LIMD1, CFLAR, PIM2, and CCND2 are common signatures associated with IRF4 in non-Hodgkin B cell lymphomas. We confirmed the correlation of IRF4 with LIMD1 and CFLAR in a panel of cell lines derived from lymphomas. Moreover, we profiled the IRF4 transcriptome in the context of EBV latent infection, and confirmed several genes including IFI27, IFI44, GBP1, and ARHGAP18, as well as CFLAR as novel targets for IRF4. These results provide valuable information for understanding the IRF4 regulatory network, and improve our knowledge of the unique roles of IRF4 in different hematological malignancies.

## Introduction

IRF4 is a lymphocyte-specific transcription factor, and its expression is confined to immune cells including B cells, macrophages, and CD11b+ DCs. IRF4 can be induced by various mitogenic stimuli which commonly activate NFκB for the activation of the IRF4 gene promoter [1]. As a transcription factor, IRF4 exerts its functions through regulation of its transcriptional targets involved in cell development, immune response, and oncogenesis. IRF4 is a quintessential ‘context-dependent’ transcription factor, and regulates distinct groups of targets in different contexts. Its DNA-binding specificity depends on lineage-specific transcriptional co-regulators. Many of these co-regulators have been identified, including two Ets family members PU.1 and SPIB, BATF, DEF6, STAT3, NFAT, FKBP52 and PGC-1α [2]–[12]. Ets and BATF are dominant co-regulators for IRF4 in B and T lymphocytes, respectively [6], [9], [11], [13]. When interacting with one of the Ets family members, IRF4, like its closest family member IRF8, binds to a composite Ets/ISRE-consensus element (EICE) with the consensus sequence 5′-GGAANNGAAA-3′, that fuses the Ets-binding motif (5′-GGAA-3′) with the IRF-binding motif (5′-AANNGAAA-3′), or to the Ets/IRF-responsive element (EIRE) with the consensus sequence 5′-GGAAANNGAAA-3′, or to the IRF/Ets-consensus sequence (IECS) 5′-GAAANN(N)GGAA-3′. When IRF4 interacts with BATF that belongs to the AP1/ATF superfamily of transcription factors, the complex IRF4-BATF binds to a composite DNA element called AICE (TGANTCA/GAAA) [14], [15].

Increasing evidence has implicated IRF4 in hematological malignancies. IRF4 overexpression is a hallmark of the activated B-cell-like (ABC) type of diffuse large B-cell lymphoma (DLBCL) and multiple myeloma (MM) [3], [13], and is also overexpressed in almost 100% cases of classical Hodgkin lymphoma (cHL), plasma cell myeloma and primary effusion lymphoma (PEL) [16]. Interestingly, high levels of IRF4 protein exist in Epstein-Barr virus (EBV)-transformed cells and associated B-cell lymphomas with Type 3 latency [17]–[20], as well as in Human T-cell Leukemia Virus-1 (HTLV1)-infected cell lines and associated Adult T-cell Leukemia (ATL) [21]–[25]. Chromosomal translocation and genetic mutation of IRF4 have been identified in MM, peripheral T-cell lymphomas [26], and Chronic Lymphocytic Leukemia (CLL) [27]. In clinical practice, IRF4 serves as an important prognostic and diagnostic marker for certain types of hematological malignancies [28]–[30]. These lines of evidence highlight the importance of IRF4 in hematological malignancies. However, only very limited transcriptional targets have been identified for IRF4 in these malignancies, and the role of IRF4 in tumorigenesis remains to be elucidated.

In this study, we aim to profile IRF4-associated unique molecular signatures in different types of hematological malignancies, by analyzing existing gene expression profile databases obtained from clinical samples. Results show that IRF4 is overexpressed in melanoma, in addition to previously reported myeoloma, lymphoma, and leukemia, and that IRF4 is associated with a unique gene expression pattern in each of these settings. Some of these genes are known IRF4 transcriptional targets, whereas some others may represent a new group of IRF4 targets. We have verified LIMD1 and CFLAR as two novel genes whose expression is correlated with IRF4 in non-Hodgkin lymphomas, and shown that CFLAR is likely an IRF4 target. Moreover, we have profiled the IRF4 transcriptome in EBV latency by using Microarray analysis and further confirmed a panel of genes including IFI27, IFI44, GBP1, and ARHGAP18 as novel IRF4 targets.

## Materials and Methods

### Datasets and Oncomine Bioinformatics

All datasets selected for this study are available on Oncomine website (www.oncomine.org). These datasets were log-transformed, median centered per array, and standard deviation normalized to one per array. Bioinformatic analysis was performed at Oncomine website, with the default parameters on the site for IRF4 co-expression analysis (threshold by p value: 10^−4^, fold change: 2, and gene rank: top 10%).

### GEO Bioinformatics

Raw data from existing Gene Expression Omnibus (GEO) datasets were downloaded. The intensity values were extracted using GeneSpring GX 12.5 software from Agilent and scaled and Quantile normalized. Values were then log transformed (base 2) to the median of all samples on the R/Bioconductor package. Two probes assigned to IRF4 gene, 204562_at and 216986_s_at, were used to divide the samples into IRF4+ and IRF4– groups. Probe 216987_at was not used as it has small variance and low expression value (mean value: 5.527 and variance 0.042). Differentially expressed genes between the two groups (IRF4+ and IRF4–) were identified using limma R package from Bioconductor with a simple two group design matrix (cut-off p≤0.05 and fold change ≥1.5). Limma is a popular library for analysis of gene expression microarray data, it uses linear model to accommodate complicated experimental design and Empirical Bayesian to provide stable results for small datasets. Benjamin-Hochberg was used for multiple testing correction.

### Cell Lines

These cell lines used in this study are well established B cell lines and have been worldwide used for EBV research for many years [31]–[34]. Akata, Sav I, Sav III, JiJoye, and P3HR1 are Burkitt’s lymphoma cell lines which were derived from patients. DG75 is an EBV-negative B cell line. IB4 and LCL.Jul are lymphoblastic cell lines *in* *vitro* transformed with EBV. All B cell lines are cultured in RPMI1640 medium plus 10% FBS and antibiotics.

### Plasmids, Reagents and Antibodies

The shIRF4 and retrovirus-mediated transfection and selection of stable transfectants were described in our previous publication [35]. Anti-LIMD1 clone H-4 (Santa Cruz), anti-FLIPs clone G-11 (Santa Cruz), anti-LMP1 CS1–4 (Dako), anti-IRF4 H140 (Santa Cruz), and anti-GAPDH (Sigma) were used for Western blotting.

### RNA Isolation, Microarray Analysis and Real-time PCR

Total RNAs from JiJoye and IB4 cell lines stably expressing shIRF4 or control were isolated using RNeasy mini kit (Qiagen) according to the manufacturer’s protocols. The eluted RNA was subjected to reverse transcriptase reactions, which were performed with the use of GoScript RT kit (Promega) following the manufacturer’s instructions. cDNAs were subjected to MOgene company (Saint Louis, MO) for Microarray analysis, with Agilent HD 4×44 k format (G4845F 026652) chips. A cross-labeling duplicate, i.e. Cy5-labeled cDNA chip for shCtl/Cy3-labeled cDNA chip for shIRF4 and Cy3-labeled chip for shCtl/Cy5-labeled chip for shIRF4, were performed for each cell line. Genes were selected with 2-fold changes as threshold.

Selected genes were confirmed by real-time PCR, as described in our previous publication [35], with specific primers. IFI27 forward: 5′-TGGCCAGGATTGCTACAGTTG-3′, and reverse: 5′-TATGGAGGACGAGGCGATTC-3′, GBP1 forward: 5′-TGGAACGTGTGAAAGCTGAG-3′, and reverse: 5′-TGACAGGAAGGCTCTGGTCT-3′, IFI44 forward: 5′- TACCAGTTTAATCCCATGGAATCA-3′, and reverse: 5′- CAAATACAAATGCCACACAATGAA-3′, and ARHGAP18 forward: 5′-AAGAGTACAAATGATGCTGACG-3′ and reverse: 5′-CCTGGCAAGTACATCACTGG-3′.

## Results

### IRF4 Expression Levels in Different Cancers

**Figure 1:**
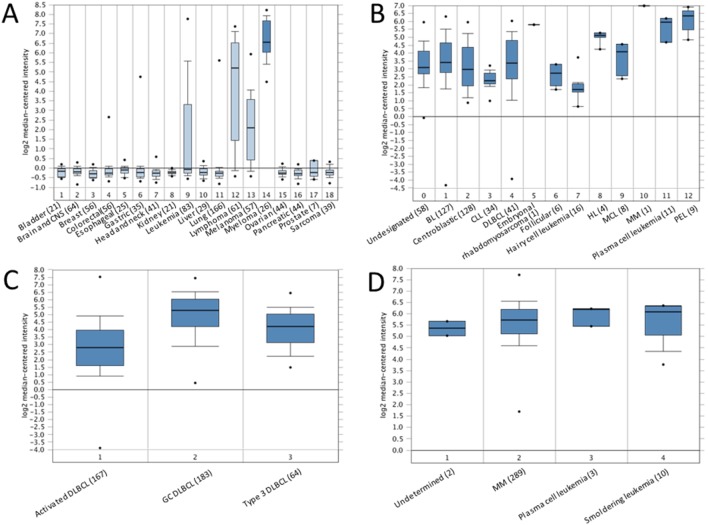
Differential expression of IRF4 in different cancer contexts. **A**. IRF4 is differentially expressed in a panel of cancer cell lines [36]. 1. Bladder cancer (21 samples); 2. Brain and CNS cancers (64 samples); 3. Breast cancer (56 samples); 4. Colorectal cancer (56 samples); 5. Esophageal cancer (25 samples); 6. Gastric cancer (35 samples); 7. Head and neck cancer (41 samples); 8. Kidney cancer (21 samples); 9. Leukemia (83 samples); 10. Liver cancer (29 samples); 11. Lung cancer (166 samples); 12. Lymphoma (61 samples); 13. Melanoma (57 samples); 14. Myeloma (26 samples); 15. Ovarian cancer (44 samples); 16. Pancreatic cancer (44 samples); 17. Prostate cancer (7 samples). 18. Sarcoma (39 samples). **B**. Differential expression of IRF4 in lymphomas [41]. 0. Undesignated (58 samples); 1. Burkitt’s lymphoma (127 samples); 2. Centroblastic lymphoma (28 samples); 3. Chronic lymphocytic leukemia (34 samples); 4. DLBCL (41 samples); 5. Embryonal rhabdomyosarcoma (1 sample); 6. Follicular lymphoma (6 samples); 7. Hairy cell leukemia (16 samples); 8. Hodgkin lymphoma (4 samples); 9. Mantle cell lymphoma (8 samples); 10. Multiple myeloma (1 sample); 11. Plasma cell leukemia (11 samples); 12. Primary effusion lymphoma (9 samples). **C**. Differential expression of IRF4 in different DLBCLs [42]. 1. Activated B cell-like DLBCL (167 samples); 2. Germinal center B cell-like DLBCL (183 samples); 3. Type 3 DLBCL (64 samples). **D**. Differential expression of IRF4 in myeloma [43]. 1. Monoclonal gammopathy of undetermined significance (2 samples). 2. Multiple myeloma (289 samples); 3. Plasma cell leukemia (3 samples); 4. Smoldering leukemia (10 samples).

We initially checked the expression levels of IRF4 in different cancer cell lines. To this end, the gene expression data, obtained from 917 cell lines in a previous study [36], was analyzed at Oncomine (http://www.oncomine.org). As shown in Fig. 1A, in 18 selected cancer types, the expression level of IRF4 was the highest in myeloma, followed by lymphoma, melanoma and leukemia. Overexpression of IRF4 has been well documented in all these cancers but only a few publications have reported the association between IRF4 genetic variants or abnormal expression and skin cancer [29], [37]–[40]. Our results have confirmed the overexpression of IRF4 in melanoma and supported the claim that IRF4 may play an important role in the development of this cancer.

Next, we analyzed the gene expression profiles for IRF4 expression in different types of B cell lymphomas with a total of 336 samples which include 36 cancer cell lines (10.7%), 125 experimentally manipulated B cell lines (37.2%), and 25 normal B cell samples (7.4%) [41]. As shown in Fig. 1B, amongst the analyzed lymphoma types, PEL has the highest level of IRF4. PEL, also known as AIDS-associated body cavity-based lymphoma (BCBL), is a malignant B cell lymphoma (non-Hodgkin lymphoma). PELs are 100% positive for Kaposi sarcoma herpesvirus (KSHV), and often contain EBV as well. Among 3 types of DLBCL (Activated, Germinal center, and Type 3), analysis results from 414 samples reveal that activated DLBCL has the highest expression level of IRF4 (Fig. 1, C) [42].

In regard to myeloma, analysis results from 304 multiple myeloma patient samples reveal that there is no significant difference of IRF4 expression levels among different stages, including smoldering (Fig. 1, D) [43].

Together, these results indicate that IRF4 is overexpressed in specific hematological malignancies including myeloma, lymphoma, and leukemia, and also in melanoma.

### Molecular Signatures Associated with IRF4 in Different Cancers

Since IRF4 is a “context-dependent” factor, it is necessary to analyze the patterns of gene expression associated with IRF4 in individual cancer types.

**Figure 2:**
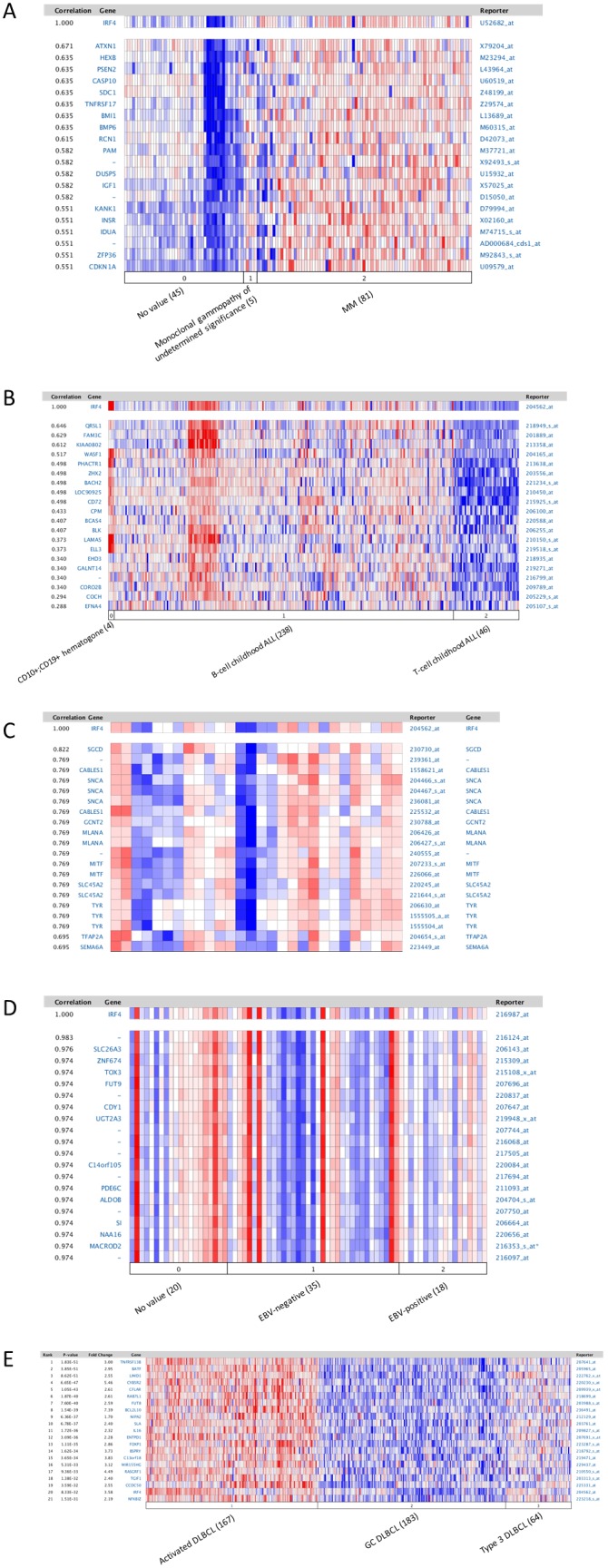
Molecular signature associated with IRF4 in hematological malignancies. **A**. IRF4-associated gene expression pattern in myeloma. The dataset includes 131 samples [44]. Analysis was performed with default parameters (p value: 10^−4^, fold change: 2, and gene rank: top 10%). 0. No value (45 samples); 1. Monoclonal gammopathy of undetermined significance (5 samples); 2. Multiple myeloma (81 samples including 74 clinical samples and 7 cell lines). **B**. IRF4-associated gene expression pattern in leukemia. The dataset includes 288 B- and T-cell acute lymphoblastic leukemia samples [45]. 0. CD10+;CD19+ hematogone (4 samples); 1. B-Cell childhood acute lymphoblastic leukemia (238 samples); 2. T-cell childhood acute lymphoblastic leukemia (46 samples). **C**. IRF4-associated gene expression pattern in melanoma. The dataset includes 28 cutaneous melanoma patient samples [46]. **D**. IRF4-associated gene expression pattern in classic Hodgkin lymphoma. The dataset includes 64 Hodgkin’s lymphoma, 3 T-cell/histiocyte-rich B-cell lymphoma, 3 Hodgkin’s lymphoma cell lines, and 3 lymphadenitis samples (60 cHL samples in total) [48]. 0. No value (20 samples); 1. EBV negative (35 samples); 2. EBV positive (18 samples). **E**. The gene expression pattern correlated with IRF4 in non-Hodgkin lymphoma DLBCL. The dataset comprises 414 DLBCL samples [42]. 1. Activated B cell-like DLBCL (167 samples); 2. Germinal center B cell-like DLBCL (183 samples); 3. Type 3 DLBCL (64 samples).

First, a pool of gene expression profile datasets obtained from different types of hematological malignancies were analyzed for genes whose expression is correlated with IRF4 using the Oncomine website. As to multiple myeloma, the dataset including 124 clinical samples and 7 cell lines (5.3%) was selected for analysis [44], with default parameters (p value: 10^−4^, fold change: 2, and gene rank: top 10%). Top 20 genes/probes correlating with IRF4 including ATXN1, HEXB, PSEN2, CASP10, are shown in Fig. 2A, and top 60 genes are shown in Table S1.

For leukemia, we analyzed the dataset which includes 288 B- and T-cell acute lymphoblastic leukemia samples [45]. As shown in Fig. 2B, IRF4 is frequently expressed in B-cell ALL, but not in T-cell ALL. Top 20 genes/probes correlating with IRF4 include QRSL1, FAM3C, KIAA0802, WASF1, BLK, ELL3, etc. Top 60 genes are shown in Table S1.

For melanoma, we analyzed the dataset which includes 28 cutaneous melanoma patient samples [46]. Top 20 genes/probes correlated with IRF4 are shown in Fig. 2C, and top 60 genes/probes are shown in Table S1. Among these genes, the transcription factor MITF, the pigmentation enzyme TYR, and MLANA and GPR143 that are involved in melanosome biogenesis, are important players in melanoma. MITF is known to cooperate with IRF4 in regulation of the expression of TYR in melanocytes [38]. Similar results were obtained from another dataset (data not shown) [47].

To analyze the molecular signature for IRF4 in cHL, the dataset including 64 Hodgkin’s lymphoma, 3 T-cell/histiocyte-rich B-cell lymphoma, 3 Hodgkin’s lymphoma cell lines, and 3 lymphadenitis samples (60 cHL samples in total), was selected for analysis [48]. As shown in Fig. 2D, overexpression of IRF4 in cHL is not dependent on EBV infection. Top 20 genes/probes include SLC26A3, ZNF674, TOX3, FUT9, etc. Top 60 genes/probes are shown in Table S1.

As to non-Hodgkin’s lymphoma DLBCL, we analyzed a dataset comprising 414 DLBCL samples [42]. Results indicate that IRF4 is expressed at a high level in activated DLBCL, along with a panel of other genes including BATF, LIMD1, CFLAR, PIM2, CCND2, IL16, miR-155, etc (Fig. 2, E and Table S1). BATF is an AP1-like transcription factor known to cooperate with IRF4 in gene transcription in B, T, and dendritic cells [6], [8], [9], [11]. Interestingly, we have previously identified the miR-155-encoding gene BIC as a target for IRF4 in viral cancer cells [35]. However, IRF4 is expressed at a lower level in Type 3 DLBCL, and at the least level in germinal center DLBCL (Fig. 2, E). Similar results were obtained with another dataset which comprises 271 DLBCL samples (data not shown) [49].

**Figure 3:**
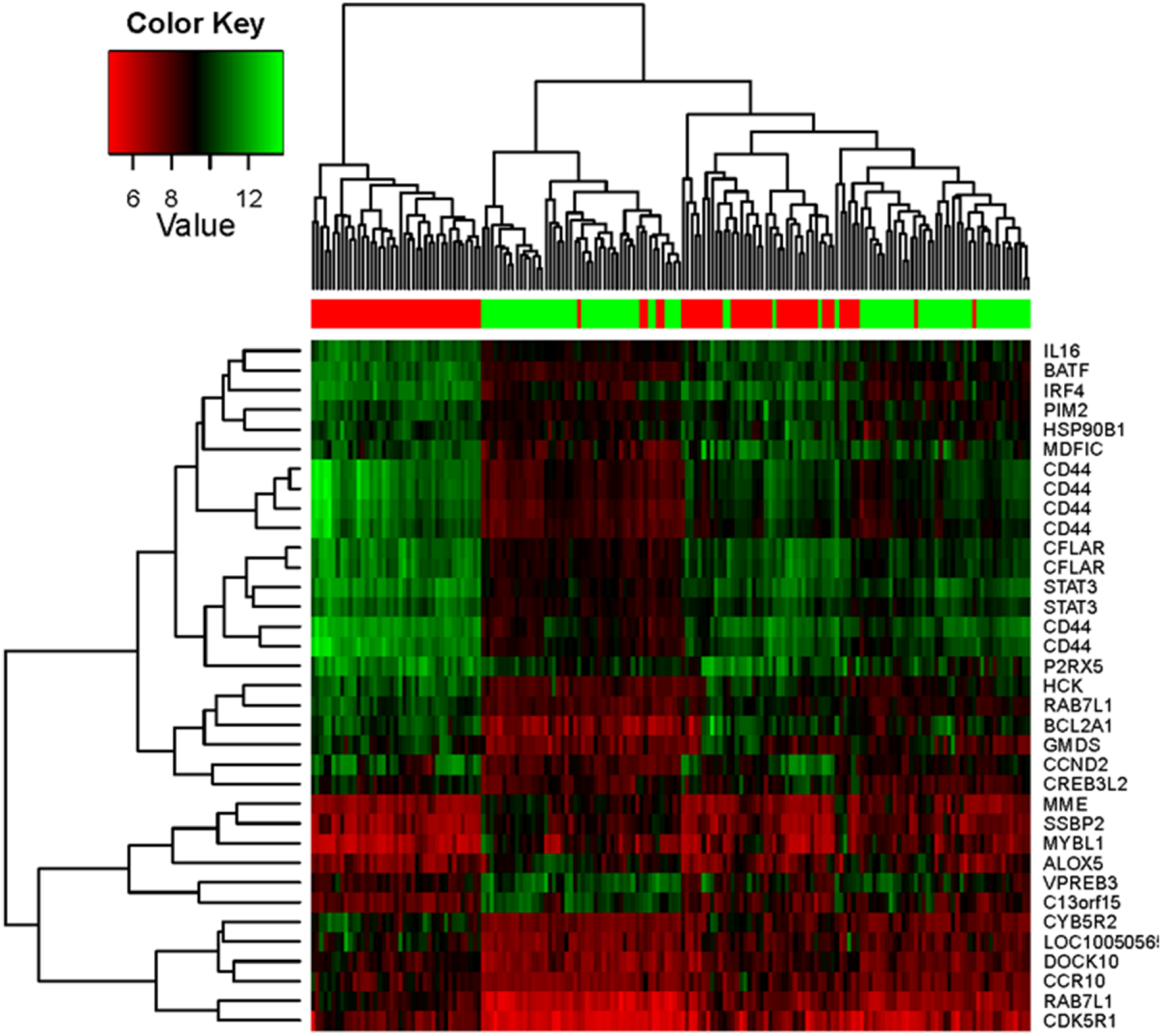
IRF4-associated gene expression pattern in lymphomas. The GEO dataset GSE4475 which includes 220 BL and DLBCL samples [50], was subjected to bioinformatic analysis for differential gene expression pattern correlated with IRF4 and gene set enrichment, as detailed in Materials and Methods. The heat map shows results from analysis with cut-off *p* value<0.01 and fold change≥2.

We also chose the GEO dataset GSE4475 which includes 220 BL and DLBCL samples [50], and performed differential expression and gene set enrichment analyses, using Limma R package and MSigDB. Our results show that, similar to other datasets from Oncomine analysis, BATF, PIM2, LIMD1, CFLAR, and CCND2, along with a panel of other genes such as STAT3, CD44, and IL16, are correlated with IRF4. Expression of some genes such as SSBP2 and MYBL1 is inversely correlated with IRF4 (Fig. 3). Results with cut-off *p* value<0.01 and fold change ≥2 are shown in Fig. 3 and Table S2. CD44 is known to be targeted by IRF4 in ABC DLBCL but not in MM [51]. Similar results were obtained from the GEO datasets GSE4732 which includes 303 Burkitt’s and B-cell lymphoma samples [52], and GSE38885 which includes 65 PTLD samples (data not shown).

Taken together, we concluded that IRF4 is associated with unique molecular signatures in myeloma, leukemia, myeloma, Hodgkin’s and non-Hodgkin’s lymphomas, and that common molecular signatures associated with IRF4 in different types of non-Hodgkin’s lymphomas include BATF, LIMD1, CFLAR, PIM2, and CCND2, amongst others.

### Verification of the Correlation of IRF4 with LIMD1 and CFLAR in EBV-associated B-cell Lymphoma

The LIMD1 (LIM domain-containing protein 1) gene is located in a chromosomal region which undergoes frequent loss of heterozygosity in many solid tumors [53]. Expression of LIMD1 is absent or decreased in many cancers including breast cancer, lung carcinoma and blood cancers [53]–[55]. LIMD1 is an adapter protein that is involved in the assembly of numerous protein complexes such as Rb [56] and TRAF6, and participates in several cellular processes such as repression of gene transcription, cell-cell adhesion, cell differentiation, proliferation and migration. LIMD1 also positively regulates microRNA (miRNA)-mediated gene silencing by binding to the core proteins of the microRNA induced silencing complex (miRISC) such as Ago1/2 [57].

**Figure 4:**
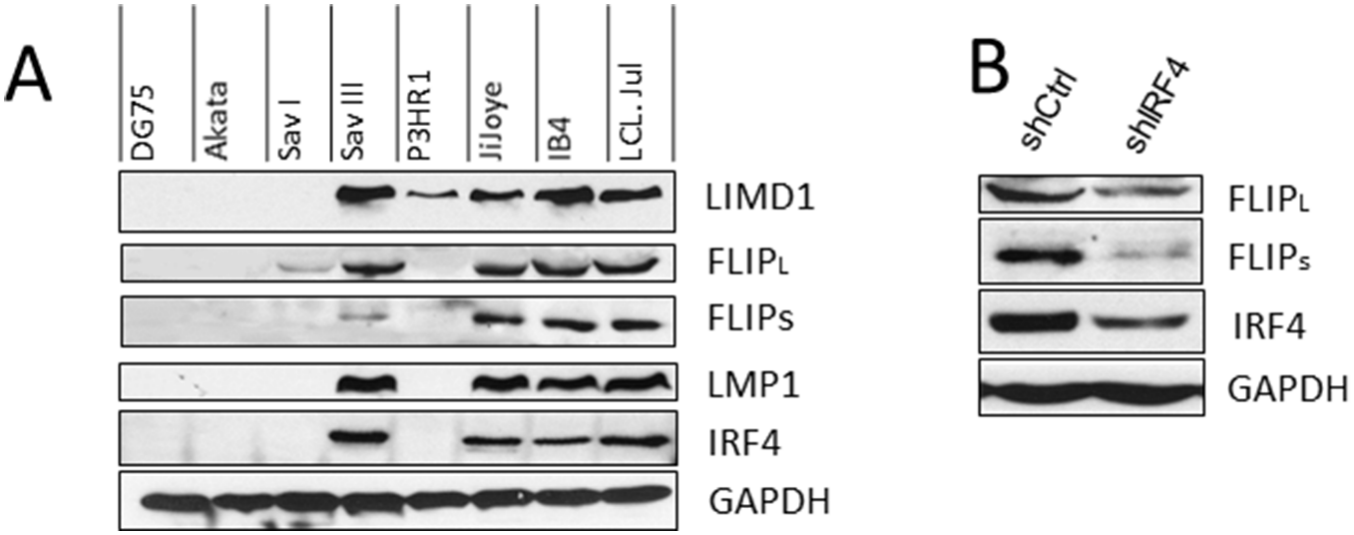
Verification of the correlation of IRF4 with LIMD1 and CFLAR. **A**. A panel of EBV-negative and -positive B cell lines were subjected for immunoblotting analysis for IRF4, LIMD1, CFLAR (c-Flip), and LMP1 expression, with specific antibodies. DG75: EBV-negative. Akata and SavI: EBV-positive with Type I latency. SavIII, P3HR1, and JiJoye were derived from EBV+ Burkitt’s lymphomas. IB4 and LCL.Jul are LCLs established in vitro by EBV transformation. **B**. Knockdown of IRF4 in JiJoye cells downregulates the c-Flip protein level in JiJoye cells.

To verify the correlation between IRF4 and LIMD1, a panel of EBV-negative and -positive B cell lines derived from B lymphomas or transformed with EBV *in* *vitro* were subjected to immunoblotting analysis with IRF4- and LIMD1-specific antibodies. As shown in Fig. 4A, LIMD1 is expressed at high levels in EBV latency 3, in which both IRF4 and LMP1 are also expressed at high levels. However, in EBV-negative and type I latency, LIMD1 is not detectable.

CFLAR encodes c-FLIP, a master inhibitor of Fas/TRAIL/TNFα-induced apoptosis [58]. Resistance to Fas/TRAIL-mediated apoptosis is a common mechanism used by tumors to evade the immune system [59]. Similar to LIMD1, CFLAR expression is high in type 3 latency, but was not detected in EBV-negative B cells and EBV type I latency (Fig. 4, A). Furthermore, depletion of IRF4 in JiJoye cells decreases endogenous CFLAR protein level (Fig. 4, B). However, depletion of IRF4 did not affect the protein level of LIMD1 (data not shown), suggesting that LIMD1 and IRF4 are both upregulated by a common transcription factor rather than LIMD1 is a direct target for IRF4.

These results confirm that IRF4 is correlated with LIMD1 and CFLAR in B lymphomas, and strongly suggest that CFLAR is a transcriptional target for IRF4.

### IRF4 Transcriptome in EBV Latency

**Figure 5:**
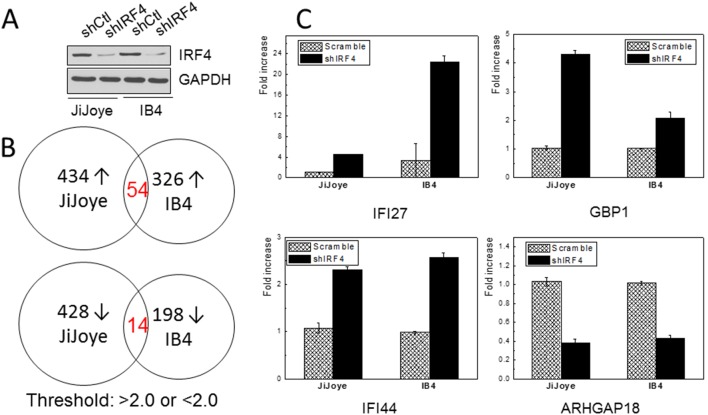
IRF4 transcriptome in EBV latency. **A**. Expression of endogenous IRF4 in JiJoye (EBV+ Burkitt’s lymphoma cell line) and IB4 (EBV+ LCL) cell lines were depleted by retrovirus-mediated transfection of shIRF4 (or control)-expressing retrovirus. Stable transfectants were selected with hygromycin and shIRF4 (or control) was induced by 1 µg/ml doxycycline. **B**. Total RNAs were extracted and subjected for Microarray analysis. Genes with >2.0 and <2.0 fold changes were selected. **C**. Real-time PCR confirmation of IRF4 transcriptional targets IFI27, GBP1, IFI44, and ARHGAP18.

IRF4 is overexpressed in EBV type 3 latency [17]–[20]. To our knowledge, BIC/miR-155 and IRF5 are the only two IRF4 targets identified in the context of EBV infection [35], [60]. To better understand the role of IRF4 in EBV latency and oncogenesis, we performed microarray analysis to profile the IRF4 transcriptome in EBV latency. Endogenous IRF4 expression in JiJoye and IB4 cells was depleted with shIRF4 as described in our previous work [35]. Knockdown efficiency of IRF4 is shown in Fig. 5, A. Total RNAs were subjected to microarray analysis. Results show that 434 genes were upregulated (>2.0 fold) and 428 were downregulated (<2.0 fold) in JiJoye cells stably expressing shIRF4 compared to cells expressing scramble control shRNA, and 324 were upregulated (>2.0 fold) and 198 were downregulated (<2.0 fold) in IB4 cells after IRF4 depletion (Fig. 5, B and Table S3).

Because these two cell lines have different genetic background (JiJoye is a BL cell line established from a BL patient and IB4 is a LCL cell line by in vitro transformation of umbilical cord B-lymphocytes with EBV), only a small portion (about 10%) of overlapping genes were identified between these two cell lines, with 54 in the upregulated gene group and 14 in the downregulated gene group (Fig. 5, B). This observation is consistent with the fact that IRF4 is a quintessential “context-dependent” factor which regulates distinct groups of targets in different contexts [5], [16].

Among the overlapping target genes between JiJoye and IB4, we confirmed the regulation of IFI27, IFI44, IFIT1, GBP1, CD88, CXCL10, and ARHGAP18 by IRF4 using real-time PCR with specific primers. IFI27, IFI44, IFIT1, CXCL10 and GBP1 are known IFN-stimulated genes (ISGs). As shown in Fig. 5C, real-time PCR results confirmed that IFI27, IFI44 and GBP1 expression levels are significantly elevated after IRF4 depletion, and ARHGAP18 level is significantly decreased (data for IFIT1, CD88 and CXCL10 are not shown). IFI27 is a mitochondrial protein involved in IFN-induced apoptosis [61], [62]. IFI44 has anti-proliferative activity and is known to be associated with HCV infection [63]. GBP1 acts as a tumor suppressor in colorectal cancer cells [64], and also plays a role in chronic active EBV infection and interacts with HCV NS5B [65]. ARHGAP18 a Rho GTPase-activating protein for RhoA and modulates cell shape, spreading, and motility [66].

Of note, CFLAR is also downregulated by shIRF4 in JiJoye cells (1.32 fold) in the microarray results, consistent with the results from GEO bioinformatics that IRF4 correlates with CFLAR in non-Hodgkin’s lymphomas and depletion of IRF4 downregulated CFLAR protein level in EBV+ lymphoma cells (Fig. 4). These data indicate that CFLAR is also a transcriptional target for IRF4. In fact, CFLAR is a known target for IRF8 [67], the closest member to IRF4 in the IRF family.

## Discussion

In this study, we have systematically profiled the molecular signatures associated with IRF4 expression in a subset of hematological malignancies including melanoma in which IRF4 is overexpressed. In addition, we have identified a panel of novel transcriptional targets including IFI27, IFI44, GBP1, CFLAR and ARHGAP18 for IRF4 in lymphomas.

Gene expression profiling datasets are available online and are convenient for identification of differential expression of a given gene and for related high throughput comparison among different cancers or under different conditions of treatments. Since a large portion of these datasets were derived from clinical patient samples, they provide an important resource which complements *in* *vitro* studies. In our analyses, the IRF4-associated molecular signatures include a subset of IRF4 transcriptional targets such as CFLAR verified in this study. Other signature genes should be co-regulated with IRF4 by upstream transcription factors such as NFκB. In future pursuits, we will combine with other strategies including cell culture systems to identify IRF4-regulated networks in individual cancer contexts.

We and others have shown that IRF4 promotes proliferation of EBV-transformed cells and IRF4 deficiency results in death of cells derived from different hematological malignancies [17], [28], [35], indicating that IRF4 plays a non-redundant role in tumorigenesis of hematological malignancies. Expression of IRF4 is associated with resistance to treatment of viral cancers with IFN/AZT, and AZT specifically induces apoptosis as well as initiates the viral lytic program in Type I EBV+ Burkitt’s lymphomas [21]. However, the mechanism of action of IRF4 accounting for this important clinical observation is unclear. Further study on the interaction between IRF4 and the IFN-inducible genes including IFI27 and IFI44 may disclose the mechanism through which IRF4 confers resistance to IFN treatment of vial cancers by regulating their expression.

Consistent with our finding that CFLAR is a transcriptional target for IRF4, CFLAR has been shown to be targeted by IRF8, the closest family member of IRF4 [67]. Correspondingly, we have identified a potential IRF4/8-binding site EICE in the CFLAR gene promoter (not shown). We will verify if this EICE is functional and responsible for IRF4/8 regulation. Targeting CFLAR by IRF4 may implicate a role for IRF4 in Fas/TRAIL/TNFα-induced apoptosis in non-Hodgkin lymphomas.

The implication of IRF4 in hematological malignancies is increasingly recognized. However, most evidence has emerged from clinical observations. Recent efforts have focused on the identification of “context-dependent” co-factors for IRF4 [2]–[11]. To date, only limited targets including miR-155 [35], IRF5 [60], Blimp1, CCNB1, BCL6, CDK6, Myc, and several others have been identified as IRF4 targets in cancers [7], [25], [28], [68]. Clearly, identification of IRF4-specific gene regulation networks will improve our understanding of its functional roles in distinct cancer contexts. Our following work will aim to disclose the roles of the novel IRF4 targets identified in our study in EBV oncogenesis.

In addition to the downstream gene regulatory networks, our recent observation that IRF4 is activated through Src-mediated tyrosine phosphorylation in EBV-associated lymphomas [69], represents another important topic and a novel challenge for studying the interaction of IRF4 with hematological malignancies. We are encouraged to identify lymphocyte-specific signaling pathways leading to IRF4 activation, which potentially opens new avenues for targeting IRF4 networks for future clinical treatments.

## Supporting Information

Table S1 **IRF4-associated molecular signatures in different hematological malignancies.**
(XLSX)

Table S2 **Bioinformatic analysis of GEO dataset GSE4475 for IRF4-assoicated molecular signatures in B cell lymphoma.**
(XLSX)

Table S3 **Microarray analysis for IRF4 transcriptome in EBV latently infected B lymphocytes.**
(XLS)
